# Urinary K^+^ promotes irritative voiding symptoms and pain in the face of urothelial barrier dysfunction

**DOI:** 10.1038/s41598-019-41971-y

**Published:** 2019-04-02

**Authors:** Nicolas Montalbetti, Sean D. Stocker, Gerard Apodaca, Sheldon I. Bastacky, Marcelo D. Carattino

**Affiliations:** 1 Renal-Electrolyte Division, Department of Medicine, University of Pittsburgh, Pittsburgh, Pennsylvania USA; 2 Department of Cell Biology, University of Pittsburgh, Pittsburgh, Pennsylvania USA; 3 Department of Pathology, University of Pittsburgh, Pittsburgh, Pennsylvania USA

## Abstract

The internal surface of the bladder is lined by the urothelium, a stratified epithelium that forms an impermeable barrier to water and urine constituents. Abnormalities in the urothelial barrier have been described in certain forms of cystitis and were hypothesized to contribute to irritative voiding symptoms and pain by allowing the permeation of urinary K^+^ into suburothelial tissues, which then alters afferent signaling and smooth muscle function. Here, we examined the mechanisms underlying organ hyperactivity and pain in a model of cystitis caused by adenoviral-mediated expression of claudin-2 (Cldn2), a tight junction protein that forms paracellular pores and increases urothelial permeability. We found that in the presence of a leaky urothelium, intravesical K^+^ sensitizes bladder afferents and enhances their response to distension. Notably, dietary K^+^ restriction, a maneuver that reduces urinary K^+^, prevented the development of pelvic allodynia and inflammation seen in rats expressing Cldn2. Most importantly, intravesical K^+^ causes and is required to maintain bladder hyperactivity in rats with increased urothelial permeability. Our study demonstrates that in the face of a leaky urothelium, urinary K^+^ is the main determinant of afferent hyperexcitability, organ hyperactivity and pain. These findings support the notion that voiding symptoms and pain seen in forms of cystitis that coexist with urothelial barrier dysfunction could be alleviated by cutting urinary K^+^ levels.

## Introduction

Histological abnormalities in the urothelium, the protective barrier that covers the interior of the bladder, have been reported in a number of pathologies that coexist with irritative voiding symptoms and pelvic pain including interstitial cystitis/bladder pain syndrome (IC/BPS), bacterial cystitis^1^, and radio/chemo-induced cystitis^2–8^. For instance, IC/BPS biopsies present high incidence of histological and microscopic features including mucosal ulcerations, urothelial ruptures, partial or completely denudated urothelium, and widening of the space between urothelial cells^3–10^. Therefore, investigators have hypothesized that urothelial barrier dysfunction allows urinary K^+^ entry into suburothelial tissues, which alters afferent signaling and smooth muscle function^11,12^. In support of this premise, clinical studies have shown that the intravesical instillation of KCl (0.4 M), also known as the potassium sensitivity test, triggers urgency and pain in ∼75% of IC/BPS patients, but not in asymptomatic controls^11,13,14^. However, because the concentration of K^+^ used in the potassium sensitivity test is significantly above the normal levels in urine (25–125 mEq/L), the question of whether urinary K^+^ contributes to symptoms generation in conditions with reduced urothelial barrier function remains open.

Urothelial barrier function depends on the presence of high resistance tight junctions (TJs)^15^, which not only regulate paracellular permeability, but may also play a role in the sensory function of the urothelium^16^. Claudins (Cldns), a group of integral membrane proteins, form the structural and functional core of TJs, and define the electrical properties of epithelia. Significantly, message for Cldn2, which forms a pore that conducts ions and water^17–19^, is upregulated at least ninety-fold in IC/BPS tissue biopsies^20^. Consistent with the notion that urothelial barrier dysfunction is sufficient to generate irritative voiding symptoms and pelvic pain, we previously showed that the acute adenoviral-mediated overexpression of Cldn2 in the rat urothelium increases urothelial permeability to ions, promotes an inflammatory process in the bladder mucosa and lamina propria, increases voiding frequency and causes pelvic allodynia (exaggerated response to touch)^21,22^.

In the current report, using dietary manipulations and a set of physiological assays we examined the mechanism by which urothelial barrier dysfunction promotes irritative voiding symptoms and pelvic pain. The results of our study indicate that in the presence of a leaky urothelium, urinary K^+^ permeates into the bladder interstitium, promoting an inflammatory process and afferent sensitization, which cause irritative voiding symptoms and pelvic pain. But, most importantly, in the face of afferent sensitization, intravesical K^+^ is necessary to perpetuate the irritative voiding symptoms.

## Results

### Urothelial barrier dysfunction reduces the mechanical threshold of bladder afferents

To investigate the contribution of urinary K^+^ to symptom generation in the face of urothelial barrier dysfunction, we used a novel rat model of cystitis induced by adenoviral-mediated overexpression in the urothelium of claudin-2 (Cldn2)^21^, a tight-junction associated protein that forms paracellular pores and is significantly upregulated in bladder biopsies from interstitial cystitis/bladder pain syndrome (IC/BPS) patients^20^. In this model, Cldn2 overexpression is restricted to the umbrella cell layer and it increases the permeability of the urothelium to ions, but not to large organic molecules^21^. Cldn2-transduced rats present with inflammation in the bladder mucosa and lamina propria, including edema and lymphocyte infiltration. Moreover, they exhibit increased voiding frequency and pelvic allodynia^21,22^.

**Figure 1:**
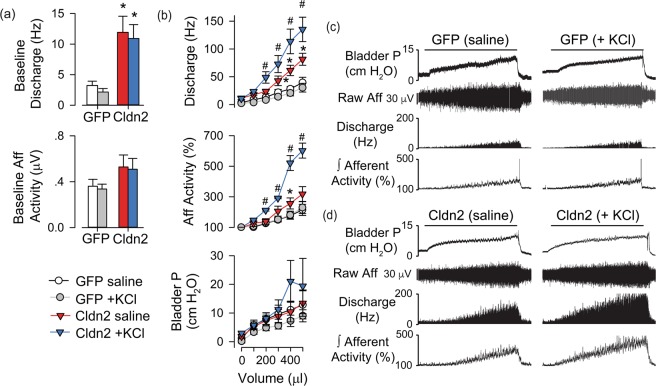
Intravesical K^+^ enhances the response of bladder afferents to distension in rats with increased urothelial permeability. Neurophysiological measurements were performed in rats transduced with replication-defective adenoviruses coding for GFP (AdGFP, n = 5) or Cldn2 (AdCldn2, n = 4). (**a**) Baseline afferent discharge (Hz) or activity (µV) of rats before intravesical infusion of saline (0.9% NaCl) or saline supplemented with 100 mM KCl (+KCl). Baseline discharge was elevated in rats transduced with AdCldn2 versus AdGFP (*p < 0.05). (**b**) Means ± SEM of afferent pelvic discharge (Hz), rectified/integrated afferent pelvic nerve activity normalized to baseline values, and bladder pressure during intravesical infusion of saline or saline supplemented with 100 mM KCl in rats transduced with AdGFP or AdCldn2. Intravesical infusion of saline or saline supplemented with 100 mM KCl produced volume-dependent increases in afferent discharge and activity in both rats transduced with AdGFP and AdCldn2. However, the response to saline was significantly greater in rats transduced with AdCldn2 vs AdGFP (*p < 0.05). Intravesical infusion of saline supplemented with 100 mM KCl exaggerated the afferent nerve response in rats transduced with AdCldn2 (#p < 0.05, Cldn2-saline vs Cldn2-KCl), but not in rats transduced with AdGFP. (**c**,**d**) Representative recordings of intravesical pressure, raw pelvic nerve afferent activity, discharge and integrated afferent activity for rats transduced with AdGFP (**c**) or AdCldn2 (**d**) and infused with saline or saline supplemented with 100 mM KCl (+KCl). A 2-way ANOVA (Group x solution) was performed to analyze differences. When significant F values were obtained, paired or independent t-tests with a layered Bonferroni correction was performed.

A hallmark of the pain and discomfort perceived by patients with IC/BPS is that it increases with bladder filling and diminishes during voiding^23–26^. To determine whether urothelial barrier dysfunction alters the mechanical threshold of bladder afferents, we measured pelvic afferent nerve activity in response to intravesical filling in rats transduced with adenoviruses coding for GFP (AdGFP) or Cldn2 (AdCldn2). Baseline, afferent discharge was significantly higher in rats transduced with AdCldn2 than AdGFP (Fig. 1a). As expected, intravesical infusion of saline increased both afferent nerve activity and discharge in rats transduced with AdGFP or AdCldn2. However, the magnitude of the change as function of the infused volume was significantly greater in rats transduced with AdCldn2 than controls (AdGFP) (Fig. 1b). To determine whether urinary K^+^ influences afferent discharge in the face of a leaky urothelium, rats were infused with saline supplemented with 100 mM KCl. Notably, afferent discharge and integrated afferent activity in rats transduced with AdCldn2 was dramatically exacerbated when bladders were distended with saline supplemented with 100 mM KCl (Fig. 1b,d), but not in those transduced with AdGFP (Fig. 1b,c). These results indicate that the presence of a leaky urothelium reduces the mechanical threshold of bladder afferents and it fosters the notion that urinary K^+^ contributes to the bladder hyperreflexia and pelvic pain seen in rats transduced with AdCldn2.

### Extracellular [K^+^] determines action potential firing of sensitized bladder sensory neurons

**Figure 2:**
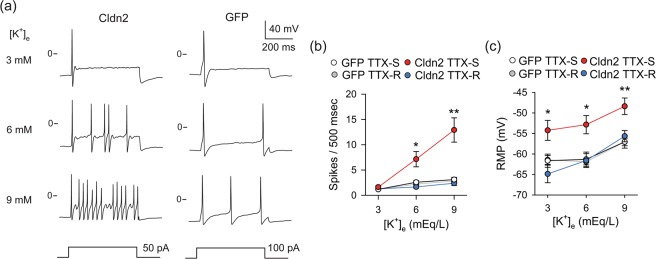
Extracellular K^+^ promotes sustained firing of sensitizes bladder sensory neurons. Bladder sensory neurons were isolated from rats transduced with replication-defective adenoviruses coding for GFP (AdGFP) or Cldn2 (AdCldn2) and cultured as indicated in Materials and Methods. Sensory neurons were classified on the basis of the sensitivity of the action potential to 1 μM tetrodotoxin (TTX), as TTX-resistant (TTX-R) or TTX-sensitive (TTX-S). (**a**) Representative tracings showing the effect of extracellular [K^+^] ([K^+^]_e_) on the firing of bladder sensory neurons with TTX-S action potentials from rats transduced with AdCldn2 (left) or AdGFP (right). Bladder neurons with TTX-R action potentials were relatively insensitive to changes in [K^+^]_e_. Current pulse protocols are shown at the bottom of the panels. (**b**) Number of action potentials evoked in response to electrical stimulation plotted as a function of [K^+^]_e_ (means ± SEM, n = 14–23,*p < 0.05 and **p < 0.01, GFP-TTX-S vs Cldn2-TTX-S, Kruskal-Wallis test ANOVA followed by Dunn’s multiple comparisons test). (**c**) Adenoviral-mediated Cldn2 expression reduces the resting membrane potential (RMP) of bladder sensory neurons with TTX-S action potentials. RMP plotted as a function of [K^+^]_e_ (means ± SEM, n = 14–23,*p < 0.05 and **p < 0.01, GFP-TTX-S vs Cldn2-TTX-S, Kruskal-Wallis test ANOVA followed by Dunn’s multiple comparisons test).

We previously showed that adenoviral-mediated expression of Cldn2 in the urothelium increases the excitability (sensitizes) of bladder sensory neurons with tetrodotoxin-sensitive (TTX-S) action potentials^22^, which are considered of Aδ origin^27^. C fibers are sensitive to capsaicin and fire action potentials that are insensitive (resistant) to tetrodotoxin (TTX-R)^28^. Under physiological conditions, the umbrella cell layer constitutes an impermeable barrier to urinary K^+^. However, in the face of a leaky urothelium, a large chemical gradient favors the diffusion of K^+^ from the urinary space into the bladder interstitium. To investigate whether changes in [K^+^]_e_ alter neuronal excitability, we labeled bladder afferents with DiI as previously described^22,28,29^. Lumbosacral (L6-S2) dorsal root ganglia (DRG) were harvested 24 h after transduction and sensory neurons were isolated and cultured as described previously^22^,^28,29^. Neuronal excitability was assessed with the perforated patch-clamp technique in the current-clamp mode^22^. To assess the effect of [K^+^]_e_ on neuronal firing, we injected a current equivalent to the rheobase for 500 ms to bladder sensory neurons bathed in extracellular solutions containing 3, 6 or 9 mEq/L of K^+^ (Fig. 2a). Action potential rheobase is defined as the minimum depolarizing current injection necessary to evoke an action potential. The number of spikes evoked in response to electrical stimulation was plotted as a function of [K^+^]_e_ (Fig. 2b). Of major significance, bladder sensory neurons with TTX-S action potentials from rats transduced with AdCldn2 display higher firing rate in bathing solutions containing 6 and 9 mEq/L K^+^ than their control counterparts (AdGFP). Consistent with our previous studies^22^, the resting membrane potential (RMP) of sensory neurons with TTX-S action potentials from rats transduced with AdCldn2 was significantly more positive than in their counterparts transduced with AdGFP (Fig. 2c). In addition, the action potential threshold was more negative and the rheobase was lower for this group (Table 1). Taken together, our findings reinforce the notion that, in the face of sensitization, even small increases in interstitial K^+^ can stimulate sustained firing of bladder sensory neurons with TTX-S action potentials.

**Table 1:** Passive and active electrical properties of lumbosacral bladder sensory neurons from rats transduced with AdGFP or AdCldn2.

	GFP		Cldn2	
	TTX-S	TTX-R	TTX-S	TTX-R
Number of cells	17	19	14	23
C_m_ (pF)	32.1 ± 1.7	31.1 ± 2.0	33.7 ± 3.3	32.1 ± 1.7
R_In_ (MΩ)	672 ± 99	708 ± 76	677 ± 123	660 ± 62
AP threshold (mV)	−26.2 ± 1.0^**#**^	−21.1 ± 0.9	−30.9 ± 0.9^#^**	−21.1 ± 0.9
Rheobase (pA)	485 ± 93	486 ± 74	246 ± 34^#^*	506 ± 76

C_m_, membrane capacitance, R_in_, input resistance, and AP, action potential. The passive and active membrane properties were determined with the perforated patch-clamp technique. Values are means ± SEM. ^**#**^p < 0.01 versus TTX-R counterparts; *p < 0.05 and **p < 0.01 versus GFP counterparts. Unpaired t test with Welch’s correction.

### Urinary K^+^ promotes referred pain in rats with increased urothelial permeability

**Figure 3:**
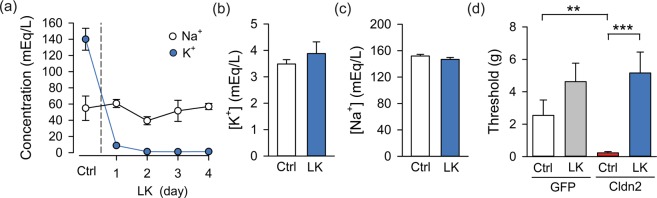
Dietary K^+^ restriction prevents the development of pelvic allodynia in rats with increased urothelial permeability. (**a**) Dietary K^+^ restriction reduces urinary K^+^ excretion. Rats fed a control K^+^ diet (Ctrl) for four days were switched to a diet with low K^+^ (LK) content. To collect urine, rats were housed in metabolic cages for 6 h at the same time of the circadian rhythm. Urinary Na^+^ and K^+^ concentrations were determined by flame photometry (means ± SEM, n = 4) as indicated in Materials and Methods. (**b** and **c**) Plasma K^+^ (**b**) and Na^+^ (**c**) concentrations for rats fed a control or LK diet for four days (means ± SEM, n = 6–7). (**d**) 50% withdrawal threshold (**g**) to von Frey filaments applied to the pelvic region for rats fed a Ctrl or LK diet and transduced with replication-defective adenoviruses coding for GFP (AdGFP) or Cldn2 (AdCldn2). Rats were subjected to dietary manipulations and three days later bladders were transduced with AdGFP or AdCldn2. Measurements were performed a day after transduction (means ± SEM, n = 15–18, **p < 0.01 and ***p < 0.001, Kruskal-Wallis test ANOVA followed by Dunn’s multiple comparisons test).

K^+^ homeostasis is maintained by adjusting renal excretion in response to variations in dietary intake. Therefore, to assess the contribution of urinary K^+^ to the pelvic allodynia seen in rats transduced with AdCldn2^22^, rats were fed a low K^+^ (LK) diet (0.0015–0.003% w/w K^+^ content, Envigo TD.88239) or a control diet (1.0% w/w K^+^ content, Envigo TD.88238) *ad libitum* with free access to water. To confirm that dietary modifications reduced urinary K^+^ excretion, rats were placed for six hours in metabolic cages at the same time of the circadian cycle for 4 consecutive days with free access to water to collect urine. To determine whether dietary K^+^ restriction alters plasma [Na^+^] or [K^+^], we collected blood from rats fed for four days a control or LK diet. Urinary and plasma [K^+^] and [Na^+^] were measured by flame photometry. As anticipated, urinary [K^+^] fell from 140 ± 13 to 8.8 ± 0.9 mEq/L, within 24 h, when rats in a control K^+^ diet were switched to a LK diet (Fig. 3a). Plasma [Na^+^] or [K^+^] were not significantly different between rats fed a control and a LK diet (Fig. 3b,c). Together, these results indicate that dietary K^+^ restriction reduces urinary K^+^ excretion without altering plasma Na^+^ or K^+^ levels.

To assess the contribution of urinary K^+^ to the pelvic allodynia seen in rats with increased urothelial permeability, we measured 50% withdrawal threshold (g) to von Frey filaments applied to the hind paw and pelvic region in rats fed a control or LK diet. Consistent with our previous report^22^, the threshold to von Frey filaments applied to the pelvic area was significantly lower in rats transduced with AdCldn2 than controls when fed a control K^+^ diet (Fig. 3d). Strikingly, rats fed a LK diet did not develop pelvic allodynia following adenoviral-mediated transduction with AdCldn2 (Fig. 3d). The 50% withdrawal threshold (g) to von Frey filaments applied to the pelvic region was comparable between rats transduced with AdGFP and those transduced with AdCldn2 fed a LK diet. No significant change in mechanical sensitivity in the hind paw was observed among the groups studied (not shown). These findings indicate that in the face of urothelial barrier dysfunction, the diffusion of urinary K^+^ into the bladder interstitium enhances afferent firing and promotes referred pelvic pain.

### Urinary K^+^ triggers the inflammatory process in rats with reduced urothelial barrier function

**Figure 4:**
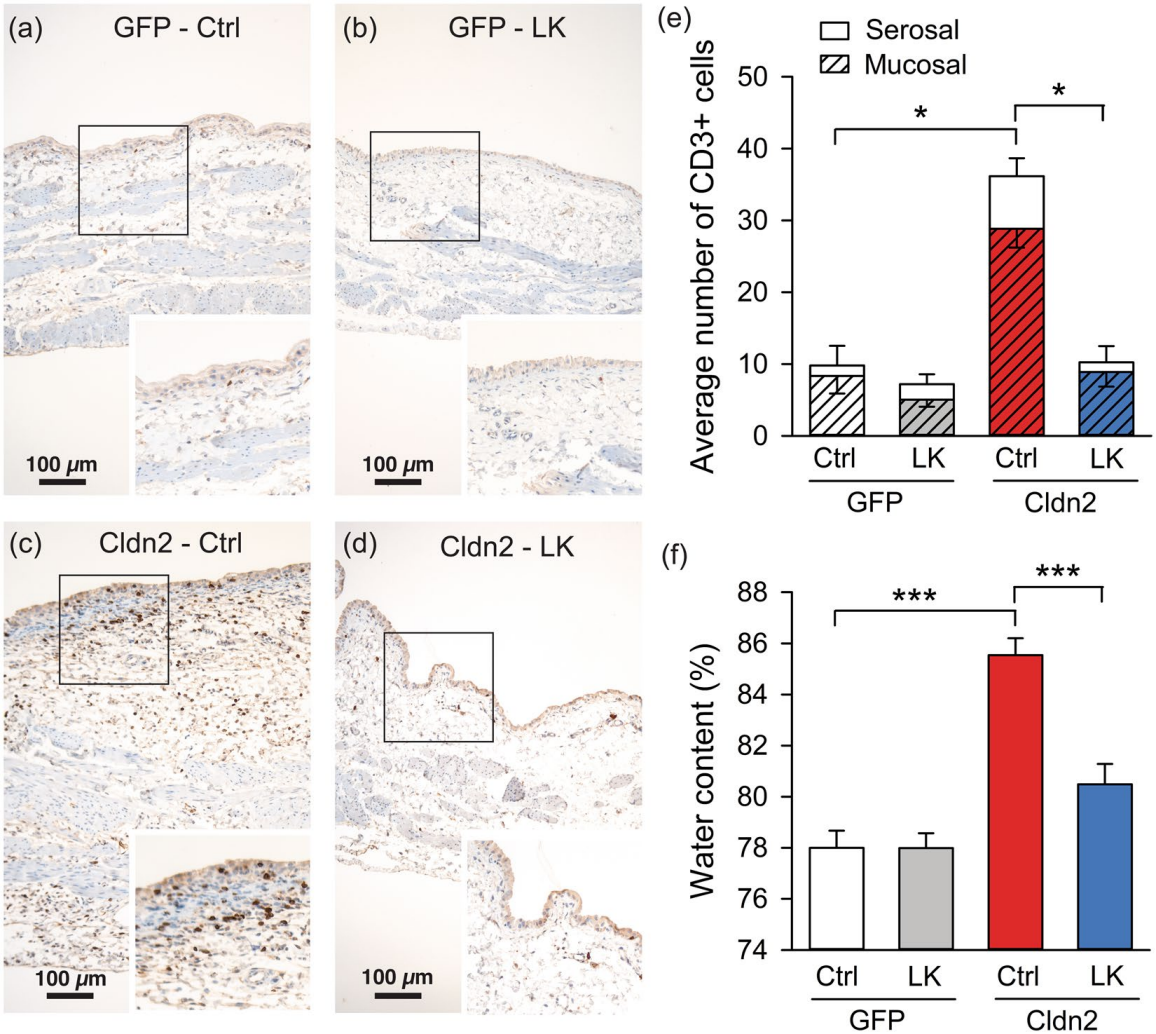
Dietary K^+^ restriction limits the inflammatory process in rats with increased urothelial permeability. A-D, CD3^+^-lymphocytes were detected by immunohistochemistry in tissues from bladders transduced with AdGFP and fed a control K^+^ diet (GFP-Ctrl, **a**) or a low-K^+^ diet (GFP-LK, **b**), or bladders transduced with AdCldn2 and fed a control K^+^ diet or (Cldn2-Ctrl, **c**) or a low-K^+^ diet (Cldn2-LK, **d**). The images show the full thickness of the bladder wall and boxed regions are magnified in the insets, which show higher magnification views of the mucosal surface of the urothelium. Note the increased number of CD3^+^-cells and increased wall thickness of bladders transduced with AdCldn2 and fed a control K^+^ diet versus the other treatments. (**e**) Average number of cross-sections through CD3^+^-cells counted in random fields of view. Cells were binned into those cells in and around the detrusor (serosal) and those in the urothelium and surrounding lamina propria (mucosal) (means ± SEM, n = 6–7, *p < 0.05, Kruskal-Wallis test ANOVA followed by Dunn’s multiple comparisons test). (**f**) Dietary K^+^ restriction limits the development of edema in rats with increased urothelial permeability. Water content of bladder determined by gravimetry for rats fed a control (Ctrl) or a low K^+^ (LK) diet and transduced with AdGFP or AdCldn2 (means ± SEM, n = 6–11, ***p < 0.001, Two-way ANOVA followed by Bonferroni multiple comparisons test).

A hallmark of IC/BPS is the presence of subepithelial inflammation and edema^9,30,31^. Consistent with the presence of an IC/BPS phenotype, we previous reported inflammatory cells, mostly lymphocytes, in the mucosa and lamina propria of bladders transduced with AdCldn2^21^. Albeit the inflammatory process, the integrity of the urothelium was preserved, and we did not observe desquamation or changes in the expression of the urothelial markers (uroplakin 3 and cytokeratin 20)^21^. Moreover, tissue water content, a measurement of edema, was significantly increased in bladders transduced with AdCldn2 when compared with controls transduced with AdGFP^22^. To assess whether dietary K^+^ restriction alters the progression of the inflammatory process in rats with a leaky urothelium, we examined paraffin-embedded bladder sections stained with H&E and the T-lymphocytic marker CD3 (Fig. 4). While bladders from rats transduced with AdCldn2 fed a control diet presented with inflammation and diffusive edema in the urothelium and lamina propria and lymphocytic infiltration (Fig. 4c), for the most part, there was little evidence of edema or inflammation in the bladders of rats transduced with AdCldn2 and fed a LK diet (Fig. 4d). The number of CD3^+^-cells counts in the mucosa and serosa of bladders from rats transduced with AdCldn2 fed a control diet was significantly greater than in rats transduced AdCldn2 fed a LK diet (Fig. 4e). No significant difference in the number of CD3^+^-cells was observed between animals transduced with AdCldn2 fed a LK diet and those transduced with AdGFP (±LK diet). In good agreement with this finding, tissue water content was significantly lower in rats transduced with AdCldn2 fed a LK diet than a control K^+^ diet (Fig. 4f). Values for animals transduced with AdCldn2, but fed a LK diet, were comparable to values for urothelium transduced with AdGFP (±LK diet). These findings indicate that urinary K^+^ plays a pivotal role in the inflammatory process triggered by the expression of Cldn2 in the urothelium.

### Intravesical K^+^ and bladder function

**Figure 5:**
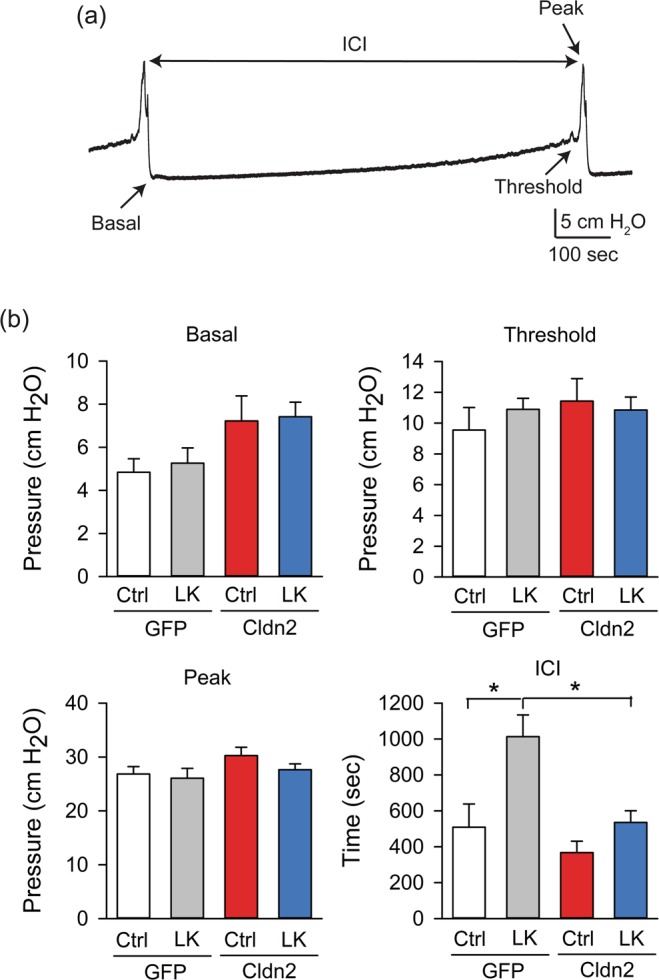
Rats expressing Cldn2 in the urothelium display normal bladder function when infused with saline. Continuous cystometry was performed in rats transduced with replication-defective adenoviruses coding for GFP (AdGFP) or Cldn2 (AdCldn2) and fed a control (Ctrl) or low K^+^ (LK) diet. (**a**) Representative cystometrogram performed in rats transduced with AdGFP and fed a LK diet. (**b**) Summary bar graphs of basal pressure, threshold pressure, peak pressure and intercontraction interval (ICI) for rats transduced with AdGFP or AdCldn2 (means ± SEM, n = 5–9, *p < 0.05, Two-way ANOVA followed by Bonferroni multiple comparisons test). A difference in basal pressure between rats transduced with AdGFP or AdCldn2 was noticed by two-way ANOVA (p < 0.05).

**Figure 6:**
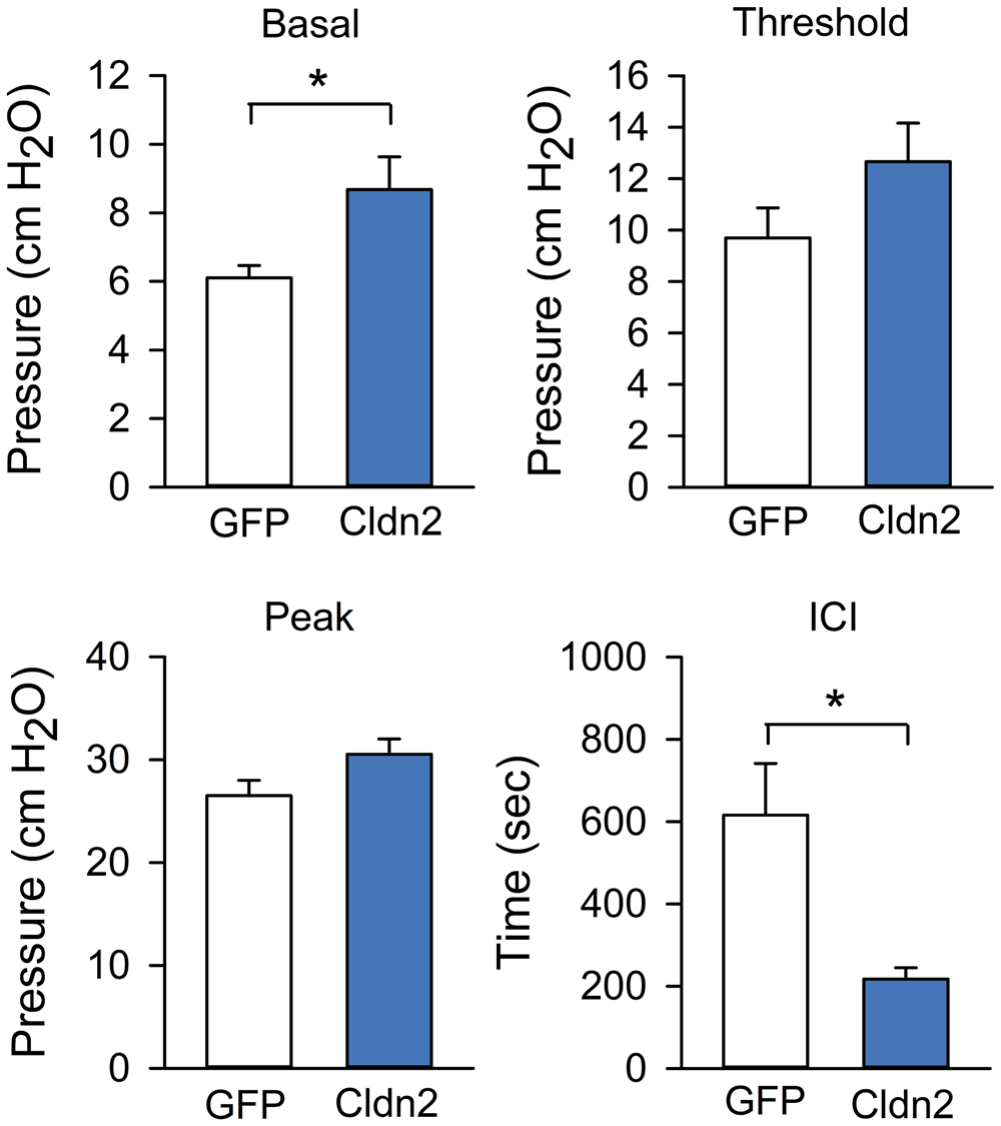
Intravesical K^+^ promotes bladder hyperreflexia in rats with increased urothelial permeability. Continuous cystometry was performed in rats fed a control K^+^ diet and transduced with replication-defective adenoviruses coding for GFP (AdGFP) or Cldn2 (AdCldn2). Rats were infused intravesically with saline supplemented with 100 mM KCl. Summary bar graphs of basal pressure, threshold pressure, peak pressure and intercontraction interval (ICI) for rats transduced with AdGFP or AdCldn2 (means ± SEM, n = 8–9, *p < 0.05, Unpair t test with Welch’s correction).

We previously reported that the presence of a leaky urothelium promotes bladder hyperreflexia by increasing the basal pressure and reducing the intercontraction interval (ICI)^21^. To assess the contribution of urinary K^+^ to the changes in bladder function seen in the face of urothelial barrier dysfunction, we performed cystometry with continuous infusion of saline (0.9% NaCl) on rats transduced with AdGFP or AdCldn2 fed a control or LK diet. A representative cystometrogram obtained 24 h after transduction with AdGFP from a rat fed a LK diet infused with saline is shown in Fig. 5a. Unexpectedly, we found that ICI was significantly longer in rats transduced with AdGFP fed a LK diet than those fed a control K^+^ diet when infused with saline (Fig. 5b). We posit that the combination of LK diet and infusion of saline during continuous cystometry alters electrolyte concentrations in the bladder interstitium and smooth muscle, causing longer ICI. No significant changes in threshold pressure, peak pressure or ICI were observed between rats transduced with AdGFP fed a control diet and AdCldn2 fed a control diet or LK diet (Fig. 5b). These findings appeared, at first, inconsistent with the hyperreflexia seen in rats transduced with AdCldn2^21^. However, for these experiments, rats were infused with saline, while in our previous studies, bladders were infused during continuous cystometry with Krebs-Ringer, which contains 5 mEq/L of K^+^ ^21^. Thus, to determine whether physiological levels of intravesical K^+^ promote bladder hyperreflexia in rats with increased urothelial permeability, rats fed a control K^+^ diet and transduced with AdGFP or AdCldn2 were infused with saline supplemented with 100 mM KCl. Consistent with our previous studies, rats transduced with AdCldn2 exhibited higher basal pressure and shorter ICI than controls (AdGFP). No significant differences in threshold pressure and peak pressure were observed between these two groups (Fig. 6).

## Discussion

The goal of this study was to define the mechanism that drives irritative symptoms and pelvic pain in a rat model of cystitis defined by urothelial barrier dysfunction. It is well known that even small changes in [K^+^]_e_ exert profound effects on neuronal excitability, smooth muscle function and inflammatory cell activation^32–40^. Because the overexpression of Cldn2 in the urothelium increases urothelial permeability to ions, we speculated that the irritative voiding symptoms and pain seen in rats transduced with AdCldn2 result from the leakage of urinary K^+^ into the bladder interstitium. Remarkably, our studies demonstrate that urinary K^+^ is the primary driver of the irritative symptoms and pelvic allodynia in the rats with increased urothelial permeability. Our findings indicate that urinary (intravesical) K^+^ acts at two stages: (i) it mediates afferent sensitization and triggers the inflammatory process in the bladder mucosa and lamina propria seen in rats transduced with AdCldn2, and (ii) it contributes to the perpetuation of the voiding symptoms. In support of the former, we showed that rats transduced with AdCldn2 fed a LK diet neither presented inflammation in the bladder nor developed pelvic allodynia. In support of the latter, we found that rats transduced with AdCldn2 fed a control diet display normal bladder function when infused with saline, but increased frequency when infused with saline supplemented with KCl. This finding indicates that even in the face of neuronal sensitization and with an undergoing inflammatory process, the removal of K^+^ from the intravesical space normalizes bladder function.

**Figure 7:**
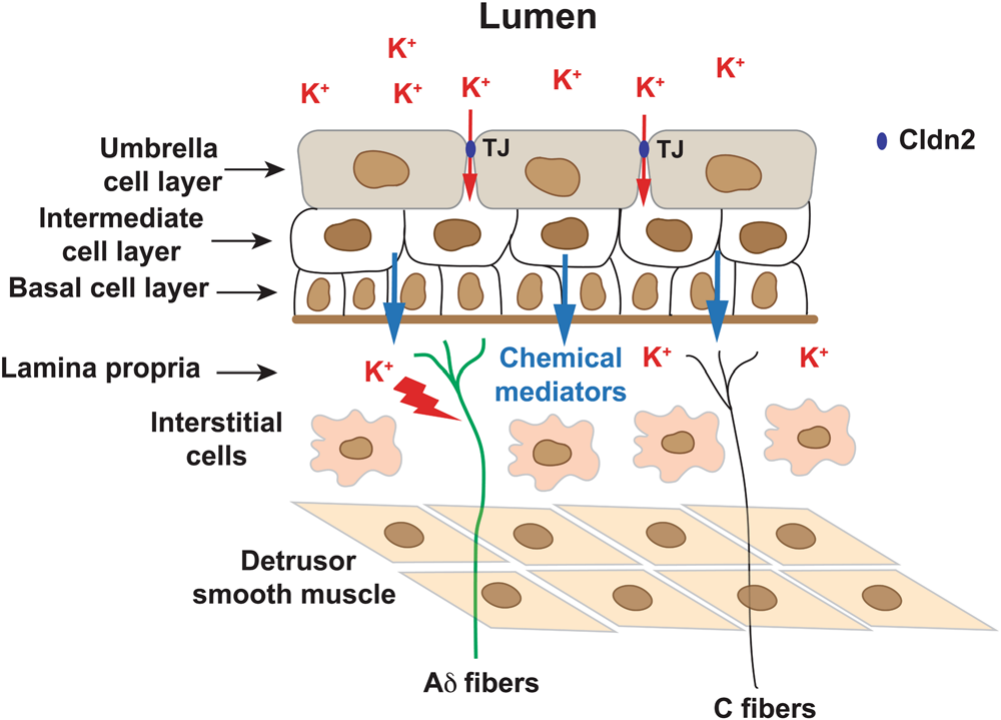
A working model of the effects of urinary K^+^ on bladder sensory signaling in the face of a leaky urothelium. Normal bladder function depends on mechanosensitive Aδ fibers that respond to bladder distention in the physiological range, while bladder nociception to a variety of chemical irritants is mediated by unmyelinated C fiber afferents. In the face of urothelial barrier dysfunction, the diffusion of urinary K^+^ into the bladder interstitium sensitizes a subset of bladder afferents with tetrodotoxin-sensitive (TTX-S) action potentials (Aδ) and triggers an inflammatory process in the bladder mucosa and serosa. Interstitial K^+^ enhances the response of Aδ bladder afferents to distension, promoting voiding symptoms and pain at low filling volumes.

How does urinary K^+^ trigger the irritative voiding symptoms and pain? We previously showed that adenoviral-mediated gene delivery of Cldn2 increases the permeability of the urothelium to ions by tenfold, but not large organic molecules^21^. Since there is a large electro-chemical gradient for K^+^ between the lumen and bladder interstitium, we posit that the overexpression of Cldn2 in the urothelium facilities the diffusion of K^+^ from the intravesical space to the bladder interstitium. We previously showed that the overexpression of Cldn2 in the rat urothelium sensitizes bladder afferents with TTX-S action potentials (Aδ). Significantly, patch-clamp studies revealed that this population of bladder sensory neurons is particularly susceptible to changes in [K^+^]_e_ and that even a modest increase in [K^+^]_e_ above physiological levels promotes aberrant firing. Multi-unit pelvic nerve recordings did not allow us to discriminate among populations of afferent fibers with TTX-S and TTX-R action potentials. However, the response of pelvic afferents to bladder distension with saline supplemented with 100 mM KCl was significantly increased in rats transduced with AdCldn2 compared to controls. As summarized in Fig. 7, our findings indicate that in the face of a leaky urothelium, the diffusion of urinary K^+^ into the bladder interstitium sensitizes Aδ afferents increasing their response to bladder distension, which promotes bladder hyperreflexia and pelvic allodynia.

Does the inflammation contribute to the irritative voiding symptoms and pain seen in rats transduced with AdCldn2? Rats transduced with AdCldn2 fed a control diet presented considerable inflammation and increased pelvic sensitivity to von Frey filaments, but normal bladder function when infused with saline. Because the removal of K^+^ from the intravesical space normalizes bladder function in the face of afferent sensitization and inflammation, we conclude that the inflammatory process does not contribute directly to the maintenance of the voiding symptoms and pain. However, we cannot rule out the contribution of the inflammation to the process of afferent sensitization in rats with reduced urothelial barrier function.

IC/BPS is a complex chronic bladder condition that presents with urinary urgency and frequency, nocturia, and pain in the bladder and/or pelvis in the absence of any identifiable cause^23–26^. Epidemiological studies revealed that more than 4 million adults have IC/BPS symptoms in the United States^33,41,42^, with an estimated female-to-male incidence of 5:1^43^. Although the etiology of IC/BPS remains unknown, an association between histopathological features and symptoms has been established by the Interstitial Cystitis Database, a multicenter study funded by the National Institute of Health (NIH) that analyzed more than 200 biopsies from patients with ulcerative (classic) and non-ulcerative IC/BPS. For example, complete loss of the urothelium was strongly correlated to nighttime voiding frequency, and pain with the percentage of mucosa denuded (8). Although the contribution of dietary K^+^ to symptom generation has not been directly evaluated in patients with IC/BPS, there is evidence that indicates that selected foods exacerbate symptoms in some patients^44^. The findings presented here indicate that foods with high K^+^ content might be an important contributing factor to symptoms generation in this condition.

In summary, our findings indicate that urinary K^+^ is a major player in symptoms generations in a model of cystitis generated by urothelial barrier dysfunction. Therefore, we conceive that the control of urinary K^+^ levels by dietary and pharmacological means might be valuable to treat voiding symptoms and pain in patients with forms of cystitis that coexist with urothelial barrier dysfunction.

## Materials and Methods

### Animals

All experimental procedures were approved by the University of Pittsburgh Institutional Animal Care and Use Committee. All methods were performed in accordance with the relevant guidelines and regulations. Female Sprague-Dawley rats (250–300 g; Envigo) were used throughout. Rats were housed under 12 h light/12 h dark cycle and fed a regular diet, a low K^+^ (LK) diet (0.0015–0.003% w/w K^+^ content, Envigo TD.88239) or a control K^+^ diet (1.0% w/w K^+^ content, Envigo TD.88238) *ad libitum* with free access to water. Animals were randomized to blinded treatment and control groups. The stage of the estrous cycle was not monitored. Rats were euthanized by CO_2_ inhalation, followed by a thoracotomy.

### Preparation of recombinant adenoviruses and *in situ* transduction of umbrella cells

Replication-defective adenoviruses coding for GFP (AdGFP) or Cldn2 (AdCldn2) were generated at the University of Iowa Viral Vector Core facility or the University of Pittsburgh Vector Core facility. *In situ* transduction of umbrella cells was accomplished *via* intravesical instillation of adenoviruses under isoflurane anesthesia (Henry Schein)^21,45^. Briefly, a day before physiological studies were conducted, a 22-gauge Teflon catheter (Smiths Medical) was inserted in the urethra to drain the urine and wash the urinary bladder. After three consecutive washes with 450 μl of Dulbecco’s phosphate buffered saline (DPBS), the bladder was infused with 450 μl of 0.1% (w/v) n-Dodecyl-β-D-maltoside dissolved in DPBS. The catheter was removed immediately after, and the urethral orifice was clamped with a metal clip to prevent leakage. The metal clip was applied to the skin and musculature surrounding the urethra. After 5 min, the catheter was reintroduced through the urethra and the bladder was emptied. The urinary bladder was infused thereafter with 450 μl of DPBS containing 2 × 10^8^ infectious viral particles (ivp) of AdGFP or AdCldn2 and the external urethral orifice was clamped again. After a 30 min incubation, the catheter was reintroduced through the urethra and the bladder was emptied and washed one time with DPBS. Then, rats were allowed to recover from anesthesia. No signs of pain due to urethral clamping (i.e. licking or scratching of the pelvic area) were apparent in the transduced animals. Experiments were performed 24 h after adenoviral transduction.

### Afferent nerve recordings

Rats were anesthetized with 2.5% isoflurane (in 100% O_2_) and instrumented with femoral arterial and venous catheters. After a tracheotomy, animals were artificially ventilated with oxygen-enriched room air. End-tidal O_2_ and CO_2_ was measured continuously and maintained at 3.5–4.5% by adjusting ventilation rate (60–90 bpm) or tidal volume (1 ml per 100 g body weight). Body temperature was maintained at 37 ± 0.2 °C by a water-blanket. Isoflurane anesthesia was replaced by urethane (1.2 g/kg body weight, IV). The left pelvic nerve was isolated, placed on bipolar stainless-steel electrodes, cut proximal, and insulated with KWIK-SIL. A heat-stretched catheter was inserted into the bladder dome for continuous infusion and intravesical pressure recordings. A second catheter was inserted into the urethra to facilitate emptying of the bladder. Nerve signals were filtered (100–1000 Hz), amplified (10,000), rectified, integrated (1 s time constant), and digitized (2000 Hz) using a micro1401 data acquisition unit and Spike2 software. In addition, action potential discharge was determined by the raw nerve signal and a window discriminator. Noise was assessed at end of recordings by cutting the nerve distal to the recording electrodes. Variables were allowed to stabilize at least 30 min after surgical procedures before any experimental manipulation. Isotonic saline (0.9% NaCl) with or without 100 mM KCl was infused at 50 μl per min (10 min) as the urethra catheter was clamped. At the end of the infusion, the clamp was released to empty the bladder. Infusions were separated by 20 min. Afferent activity was analyzed in 10 sec bins every 30 sec during the experiment. Baseline values represent an average of 10 sec bins over a 3 min baseline period immediately before the infusion.

### Retrograde labeling of bladder sensory neurons

Bladder afferent neurons were labeled with the fluorescent dye DiI (1,1′-dioctadecyl-3,3,3′,3′-Tetramethylindocarbocyanine perchlorate, Invitrogen) as reported previously^22,28,29^. Briefly, rats were anesthetized with isoflurane and the bladder was exposed through an abdominal incision (∼1–2 cm in length). Dil (5% w/v in DMSO) was injected at three to four sites (total volume, 20–30 μl) in the bladder wall. At each injection site, the needle was kept in place for 20–30 sec after inoculation to prevent leakage. Any visible leakage of dye was removed by application of a cotton swab and rinsed with saline. The muscle layer and skin incision were individually closed with 5.0 PDO absorbable monofilament surgical suture (AD Surgical). Postoperative analgesia was provided by subcutaneous administration of ketoprofen (5 mg/kg) (Zoetis). Ampicillin (10 mg/kg) (Boehringer Ingelheim Vetmedica) was administrated to prevent infections. Rats were housed under the conditions described above between 9–15 days before any further procedure was performed.

### Isolation of bladder sensory neurons

Lumbosacral (L6-S2) dorsal root ganglia (DRG) collected from a rat injected with DiI were transferred to a cell culture dish containing neurobasal media (Neuro-A medium supplemented with 5% of B27 supplements, 0.5 mM L-glutamine, and 100 U/ml of penicillin and 100 ug/ml of streptomycin, Invitrogen). DRG were minced and agitated in a cell culture flask with 5 ml of neurobasal media supplemented with 10 mg of collagenase type 4 (Worthington Biochemical Corporation) and 5 mg of trypsin (Worthington Biochemical Corporation) for 30 min at 37 °C. Tissue fragments were gently triturated with a fire-polished glass pipette and the cell suspension was centrifuged at 420 g for 5 min. The pellet, containing DRG somas, was resuspended in neurobasal media, and the centrifugation and resuspension steps were repeated three times. The pellet from the last centrifugation was resuspended in 1.5 ml of neurobasal media and the suspension was plated on coverslips coated with ornithine (Sigma) and laminin (Invitrogen) inside a 6-well tissue culture plate. After an incubation of 2 h at 37 °C with 5% CO_2_, 3 ml of warm neurobasal media was added to each well and the tissue culture plate was returned to the incubator. Electrophysiological studies were performed within 2 and 10 h of plating.

### Patch-clamp studies

Whole cell patch-clamp recordings from isolated bladder sensory neurons were obtained with the perforated technique using Amphotericin B. Current-clamp recordings were performed at room temperature with an Axopatch 200B patch-clamp amplifier (Molecular Devices) and data were captured with a Digidata 1440 A acquisition system and pClamp 10 (Molecular Devices). Signals were low-pass filtered at 1 kHz (four-pole Bessel filter) and digitized at 5 kHz. Micropipettes were pulled from borosilicate glass capillary tubes (Warner Instruments) with a PP-830 puller (Narishige). Fire-polished micropipettes with a tip resistance of 1.5–3 mΩ were used for patch-clamp recordings. The pipette filling solution contained (in mM): 145 KCl, 1 MgCl_2_, 0.1 CaCl_2_, 1 EGTA, and 10 HEPES (pH 7.2). Amphotericin B was added to the pipette solution to a final concentration of 120 μg/ml. The extracellular bath solution contained (in mM): 135 NaCl, 5 KCl, 1 MgCl_2_, 2.5 CaCl_2_, 10 glucose and 10 HEPES pH 7.4. A gravity-feed perfusion system (Automate Scientific) was used to exchange solutions.

### Patch-clamp data analysis

Patch-clamp experiments were analyzed with Clampfit (Molecular Devices). All the neurons studied showed a resting membrane potential more negative than −40 mV and generated action potentials with a distinct overshoot higher than 0 mV in response to depolarizing current injections. The input resistance was calculated from the slope of current/voltage relationships generated by injecting a series of 400 msec current pulses from −75 to 50 pA in steps of 25 pA. Action potential rheobase, defined as the minimum depolarizing current injection necessary to evoke an action potential, and action potential threshold, defined as the maximum membrane potential depolarization obtained in the absence of an action potential, were determined by injecting a series of 4 msec rectangular depolarizing current pulses of increasing intensity. To examine firing patterns in bladder sensory neurons, a 500 msec depolarizing rectangular current pulse equivalent to the rheobase was injected and the number of spikes was calculated.

### Determination of urinary and plasma [Na^+^] and [K^+^] by flame photometry

To collect urine, rats were housed in individual metabolic cages (Tecniplast) for 6 hours with free access to water. The collection container was filled with 1 ml of mineral oil to prevent evaporation. Blood was collected by cardiac puncture using a heparinized syringe. Blood was centrifugated for 5 minutes at 1,500 × g, plasma was collected and store in a microcentrifuge tube. Urine and plasma samples were frozen until further analysis. [Na^+^] and [K^+^] were determined with a flame photometer model PFP-7 (Buck Scientific) using a mixture of air/natural gas. Urine and plasma samples were diluted in deionized water as recommended by the manufactured. Standards were prepared by serial dilutions of a stock with define amounts of Na^+^ and K^+^ (Nova 4 microsample standard, Nova Biomedical). Samples were measured in duplicate and [Na^+^] and [K^+^] estimated by interpolation of the reading values with the standard curve.

### Assessment of mechanical allodynia

Thresholds to von Frey filaments applied to the pelvic area and hind paw were estimated with the up-down method described by Chaplan and colleagues^46^. Rats were placed in modular cages (Bioseb) on an elevated wire-mesh platform to allow access to the pelvic area and plantar surface of hind paw. The animals were acclimatized for at least 1 h prior to the test. von Frey filaments were applied on the plantar surface of a hind paw and on the lower abdominal area close to the urinary bladder for 1–3 sec with intervals between stimuli of 15 sec. Testing in the pelvic area was initiated with a von Frey filament with a calibrated force of 1 g. Testing in the hind paw was initiated with a von Frey filament with a calibrated force of 10 g. When a negative response was observed, the next-stronger filament was applied. When a positive response was observed, the next-weaker stimulus was applied. Abdominal withdrawal (either contraction of the abdominal musculature or postural retraction of the abdomen), licking or scratching in the pelvic area in response to von Frey filament application were considered a positive response. When stimuli were applied to the plantar surface, a response was considered positive when the animal withdrew the paw sharply or licked the tested limb. After the response threshold was first crossed, four additional filaments were applied that varied sequentially up or down based on the animal response. The resulting pattern of positive and negative responses was tabulated and the 50% response threshold was calculated using the equation:

$$50 \% \,{\rm{g}}\,{\rm{threshold}}=\frac{{{\rm{10}}}^{{[{\rm{X}}}_{{\rm{f}}}+{\rm{k}}{\rm{\delta }}]}}{\mathrm{10},\mathrm{000}}$$

where X_f_ represents the value of the final von Frey filament used, *k* represents the tabular value for the pattern of positive/negative responses, and *δ* represents the mean difference (in log units) between stimuli^46^.

### Tissue edema quantification

Urinary bladders were harvested through an abdominal incision, carefully dried with a filter paper to eliminate the urine and weighed to obtain the wet mass (WM). To determine the dry mass (DM), samples were placed in an incubator at 55 °C until constant weight was reached. The percentage of water tissue content was calculated as,

$$\frac{{\rm{WM}}-{\rm{DM}}}{{\rm{WM}}}\times 100$$

### Quantitation of CD3^+^ cells

Bladders transduced with AdGFP or AdCldn2 were cut into 5–7 random strips, paraffin-embedded sections (5 µm in thickness) that included sections through all strips were mounted on slides, and then processed for immunohistochemistry with a BenchMark Ultra slide processor with an IHC/ISH staining module (Roche) and a CONFIRM anti-CD3 (2GV6) rabbit monoclonal primary antibody (Roche) according to the manufacturer instructions. Subsequent analysis and counting were performed in a double-blind manner. Each slide (containing transects through each of the bladder’s strips) was placed on the stage of a Leica DM6000B upright microscope (fitted with a 20X HC PL-APO, 0.8 N.A., objective), and the number of CD3^+^-cells was counted in 1–3 random image fields from each of the 5–7 strips taken from each bladder. The average number of images per bladder was ~10, and for each treatment group there were 6–7 bladders. Thus, we counted ~60–70 random images for each treatment group. Counts were binned into two groups: CD3^+^-cells in the urothelium and lamina propria (mucosal), and CD3^+^-cells in the connective tissue surrounding the detrusor and serosa (serosal). For each bladder, the data from each of the 5–7 slices was used to calculate an average number of CD3^+^-cells per field. For publication, images were captured using a Jenoptic Progres Graphyx Prokyon color digital camera interfaced with an Apple iMac computer running Progres Graphyx image capture software. Exported images were opened in Adobe Lightroom CC 2018 and adjustments made to the white point, clarity, and exposure. The corrected images were then exported in TIFF format and compiled in Adobe Illustrator CC 2018.

### Assessment of bladder function by continuous infusion cystometry

Rats were anesthetized with urethane (1.2 g/Kg) and their bladders were exposed through an abdominal incision. Upon being exteriorized, shallow purse string sutures were made around the dome using a 6–0 silk suture. The area within the boundary of the sutures was punctured with an 18-gauge needle, and a flame-flanged PE-50 tube was inserted into the hole. The suture was tightened around the tubing and the tube was gently retracted until the flange was flush with the mucosa. The bladder was returned to the peritoneal cavity and the surgical incision was closed. The PE-50 tubing was connected to a three-way port: one branch led to a pressure transducer (ADInstruments), while another was connected to a syringe pump for continuous infusion with saline or saline supplemented with 100 mM KCl. The pressure transducer was connected to a Quad Bridge Amplifier and Powerlab 4/30 (ADInstruments), which was interfaced to a computer running the program Chart 8.0 (ADInstruments). Solutions were infused into the bladder at a rate of 35 μl/min. Data for successive bladder voiding cycles were collected and analyzed as previously described^47^. For each animal the following parameter were estimated: (1) basal pressure, the lowest pressure recorded after a void; (2) threshold pressure, the pressure recorded just before voiding was triggered; (3) peak pressure, the maximum pressure recorded during voiding; and (4) intercontraction interval (ICI), the time between two consecutive voids. The values of basal pressure, threshold pressure, peak pressure, and ICI for each animal represent the average from at least four data points.

### Statistics

Data are expressed as the mean ± SEM (*n*), where *n* equals the number of independent experiments. To compare differences between two groups we used unpair t test with Welch’s correction, which does not assume equal standard deviations. Two compare more than two groups, the equality of the variances was tested with Bartlett’s test. If the variances were not significantly different, when to independent variables were compared, two-way ANOVA followed by Bonferroni multiple comparisons test was used to analyze differences between groups. When the variances were significantly different, data were compared with Kruskal-Wallis ANOVA test followed by Dunn’s multiple comparisons test. *p* < 0.05 was considered statistically significant. Fitting and statistical comparisons were performed with Clampfit (Molecular Device, Sunnyvale, CA), Sigmaplot 12.5 (Systat Software, Chicago, IL) or GraphPad 7 (GraphPad Software, San Diego, CA).
